# Editorial: Spotlight on the Relationship Between Sepsis and Infection: From Mechanisms to Therapy

**DOI:** 10.3389/fmed.2021.716215

**Published:** 2021-08-02

**Authors:** Alessandro Russo, David L. Paterson, Matteo Bassetti

**Affiliations:** ^1^Infectious and Tropical Disease Unit, Department of Medical and Surgical Sciences, “Magna Graecia” University of Catanzaro, Catanzaro, Italy; ^2^University of Queensland Centre for Clinical Research, The University of Queensland, Brisbane, QLD, Australia; ^3^Infectious Diseases Unit, Ospedale Policlinico San Martino - Istituto di Ricovero e Cura a Carattere Scientifico, Department of Health Sciences (DISSAL), University of Genoa, Genoa, Italy

**Keywords:** sepsis, septic shock, infection, antimicrobial therapy, multi-drug resistant pathogens

## Introduction

The dysregulated host response to infection leading to sepsis and septic shock is a life-threatening event that, despite advances in organ support and antimicrobial therapy, is associated with a mortality rate of >30% (1). Despite the implementation of international guidelines supporting early-goal-directed therapy, recent randomized trials have demonstrated that these interventions do not improve the survival of septic patients. This evidence warrants an urgent clarification of the molecular mechanisms underlying clinical responses in patients with sepsis or septic shock. Therefore, there is an urgent global need to improve the prevention, recognition, diagnosis, and management of sepsis. The key to improving these processes lies in acquiring in-depth knowledge of the intricate interplay between host defense, infection, and pathogen virulence as well as the timing and type of interventions that are most effective according to the personal characteristics of individual patients. In recent years, infections due to multidrug-resistant (MDR) Gram-negative and Gram-positive pathogens, have been increasingly observed among critically ill patients admitted to the intensive care unit, but also in medicine wards. In this scenario, a predominant role of fungal etiology was associated with the development of severe infections (2).

The management of critically ill patients includes early diagnosis and immediate administration of antimicrobials (3–5). Previous observations about septic patients highlighted the crucial role of timely empirical antimicrobial treatment and the importance of a definitive anti-infective therapy with *in vitro* activity against the microbial isolates, emphasizing also the importance of adequate and early source control of infection (6–8). Moreover, the pharmacokinetic and pharmacodynamic properties of antibiotics should be considered due to changes in the clearance and volume of distribution that are frequently observed in critically ill patients, with the potential to influence the concentration of the drug at the site of infections (9, 10).

Recently, new drugs for the treatment of severe infections have been approved but their role in clinical practice needs to be focused (11–15). As mentioned previously, knowledge of mechanisms related to progression from sepsis to septic shock and adequate management of patients, including choice and dosages of antimicrobials, prove crucial to improving the outcome of septic patients (16).

In this Research Topic, the authors have provided contributions about crucial aspects of sepsis: (1) the key roles of mechanisms of sepsis susceptibility, clinical presentation, and outcomes; (2) new avenues for healthcare intervention and to accelerate improved treatments for sepsis; (3) a specific focus on MDR etiology and new antimicrobial therapies.

## Mechanisms Underlying Sepsis

Lonsdale et al. discussed the pathophysiology underlying sepsis and inflammatory response, reviewing the current management strategies. In the review by Jarczak et al., the authors provided an overview of sepsis immune pathophysiology, to update the choice of therapeutic approaches targeting different immunological mechanisms in the course of sepsis and septic shock, and to call for a paradigm shift from the pathogen to the host response as a potentially more promising approach.

Fenner et al. analyzed a new syndrome, chronic critical illness, that includes sepsis patients who survive the early “cytokine or genomic storm,” but fail to fully recover, and progress into a persistent state of manageable organ injury requiring prolonged intensive care. Despite being a common outcome, there are no therapeutic interventions other than supportive therapies for this common disorder. Only through an improved understanding of the immunological endotype rational therapeutic interventions could be designed.

Chung and Claus focused their review on the role of acid sphingomyelinase (ASM) that when secreted can hydrolyze sphingomyelin present at the outer leaflet of membranes to ceramide. During an episode of sepsis, a broad panel of cells, tissue, and organ response is controlled by stress-induced ceramide generation. There is a broad understanding of the potentially harmful effects of ceramide generation during sepsis and there is a wide range panel of well-established and approved drugs with effectiveness for ASM inhibition, indicating the need for more systematic studies for detailed examination of an unattended or an intended inhibition of ASM during sepsis to improve patient outcomes.

Zetoune and Ward highlighted the role of complement activation products (especially C5a anaphylatoxin and its receptors C5aR1 and C5aR2) on the adverse effects of sepsis. During sepsis, the appearance of these complement products is followed by the appearance of extracellular histones in plasma, which have powerful proinflammatory and prothrombotic activities that cause cell injury and multiorgan dysfunction in septic mice, but also in septic humans. Histone appearance in plasma is related to complement activation and the appearance of C5a and its interaction with its receptors. Neutralization of C5a with antibody or absence of C5aR1 blocks the appearance of extracellular histones and cell and organ failure in sepsis, with survival rates in septic mice greatly improved after blockade of C5a with antibody.

## Risk Factors for Sepsis and Septic Shock

Jin et al. described the clinical and microbiological characteristics and mortality predictive factors in patients with bloodstream infections (BSI). A higher mortality rate was significantly associated with older age, cancer, sepsis diagnosis, intensive care unit (ICU) admission, and prolonged hospital stay prior to BSI onset.

Banerjee et al. highlighted the emergence and burden of XDR hypervirulent isolates of *K. pneumoniae*, causing neonatal sepsis in a tertiary care hospital in India. In another study, Silveira et al. analyzed the genetic basis of antimicrobial resistant Gram-negative bacteria isolated from bloodstreams in Brazil. These data may help physicians manage patients with BSI or other nosocomial infections caused by these pathogens. The study by Mukherjee et al. discusses the current understanding of neonatal sepsis caused by carbapenem-resistant *Klebsiella pneumoniae* (CRKP), including hypervirulent *Klebsiella pneumoniae* in neonates, with a focus on strategies to effectively identify and treat these organisms.

Tompkins et al. reported data about antibiotic resistant Enterobacterales in Sub-Saharan Africa. Genes causing antibiotic resistance are easily spread between the environment and humans and infections due to drug resistant organisms contributing to sepsis mortality *via* delayed time to appropriate antimicrobial therapy. Additionally, second or third-line antibiotics are often not available or are prohibitively expensive in resource-constrained settings, leading to limited treatment options. Lack of access to water and sanitation facilities, unregulated use of antibiotics, and malnutrition are contributors to high rates of antibiotic resistance in the region. The authors concluded that improvements in the monitoring of drug resistant infections and antibiotic stewardship are needed to preserve the efficacy of antibiotics for the future.

Finally, Gudiol et al. reviewed the mechanisms involved in the development of sepsis and septic shock in patients with cancer, focusing on the risk factors associated with a worse prognosis, the impact of adequate initial empirical antibiotic therapy, and the optimal management of sepsis in this special population.

## Advances in Diagnosis

Giacobbe et al. focused on the role of machine learning techniques to early detect sepsis. A rigorous multidisciplinary approach to enrich our understanding in the application of machine learning techniques for the early recognition of sepsis may show potential to augment medical decision-making when facing this heterogeneous and complex syndrome.

Banerjee and Humphries described new and emerging phenotypic and genotypic antimicrobial susceptibility testing methods and summarized the evidence that implementation of these methods can impact clinical outcomes of patients with BSI.

In patients with complicated infective endocarditis (IE) Lin et al. observed that a high SOFA score, combined with increased CRP levels, was associated with in-hospital mortality. Moreover, the SOFA score predicted long-term mortality in complicated IE. In the review by Cuervo et al., the authors report on current challenges in the management of IE that require the close collaboration of multidisciplinary endocarditis teams.

In the study by Meini et al., D-dimer resulted in a useful tool to stratify the risk of in-hospital mortality and complications in patients with invasive infections due to *Neisseria meningitidis*, while for *Streptococcus pneumoniae* the mortality rate was irrespective by D-dimer values.

Thy et al. analyzed the microbiological characteristics of surgical samples obtained during initial surgery, compared with those obtained during the first reoperation. The authors concluded that the emergence of microorganisms, including MDR bacteria, is frequently noted in necrotizing skin and soft tissue infections, however without affecting mortality.

Interestingly, Huang et al. discussed the role of Fission1 (Fis1) and parkin as key proteins related to mitochondrial fission and mitophagy, respectively. In this study the Fis1/parkin ratio resulted in being valuable for risk stratification in patients with sepsis and is associated with poor clinical outcomes for sepsis in the ICU.

Claus and Graeler reviewed the role of mass spectrometry of sphingolipids and related species (“sphingolipidomics”) to investigate the cellular and compartment-specific response to stress in sepsis. The authors concluded that this method is on the rise and the ability to integrate multiple datasets from diverse classes of biomolecules by mass spectrometry measurements and metabolomics will be crucial to fostering our understanding of human health as well as response to disease and treatment.

Finally, the potential role of nasopharyngeal microbiome (NP) in *Streptococcus pneumoniae* invasive infection (IPD) was reviewed by Dietl et al. Although NP microbiome in patients with IPD has not been properly characterized yet, there seem to be discordant results between pediatric and adult populations; then, new longitudinal studies with a larger number of participants and a homogeneous system to collect samples should help to elucidate the potential role of the previously observed microbial species in adults and their relationship with increasing or reducing risk for the development of respiratory infections, especially IPD.

## Management, Antimicrobials, and Adjunctive Therapies

Kimmig et al. review and discuss current diagnostic and therapeutic key elements and open questions for the management of Staph*ylococcus aureus* BSI.

Hites discussed the different anti-infectious treatments currently available and suggestions on how to deliver optimized dosage regimens to septic patients, with a particular emphasis on newly available anti-infectious therapies. In the review by Corcione et al., the authors focused on the role of novel cephalosporins in septic subjects and severe infections, reporting present findings and future perspectives.

Busani et al. reported on how adjuvant therapies can help physicians to modulate the immune system, summarizing the rationale for using immunoglobulins as an adjunctive treatment.

The study of d'Ettorre et al. reports on a specific bacterial formulation that showed a significant ameliorating impact on the clinical conditions of patients with SARS-CoV-2 infection, focusing on the role of complementary therapeutic strategy to avoid the progression of COVID-19.

## Conclusion

In this Research Topic, the authors focused on crucial aspects of sepsis, including the mechanisms underlying sepsis, risk factors for the development of sepsis and septic shock, advances in diagnosis, management, and therapy of these severe infections, and the role of adjunctive therapies for the treatment of septic patients. The management of septic patients includes early diagnosis and immediate administration of antimicrobials through the identification of risk factors and mechanisms underlying the development of sepsis and septic shock. A timely empirical antimicrobial treatment and the importance of definitive anti-infective therapy with *in vitro* activity against the microbial isolates are crucial to improve survival, emphasizing the importance of an adequate and early source control of infection. Moreover, the pharmacokinetic and pharmacodynamic properties of antibiotics should be considered because of changes in clearance and volume of distribution that are frequently observed in critically ill patients, with the potential to influence the concentration of the drug at the site of infections.

## Author Contributions

AR planned the editorial. DP and MB performed revision of the text. All authors contributed to the article and approved the submitted version.

## Conflict of Interest

The authors declare that the research was conducted in the absence of any commercial or financial relationships that could be construed as a potential conflict of interest.

## Publisher's Note

All claims expressed in this article are solely those of the authors and do not necessarily represent those of their affiliated organizations, or those of the publisher, the editors and the reviewers. Any product that may be evaluated in this article, or claim that may be made by its manufacturer, is not guaranteed or endorsed by the publisher.
